# Supplementary material for: Role and mechanism of NCAPD3 in promoting malignant behaviors in gastric cancer
Source: Front Pharmacol. 2024 Apr 22;15:1341039. doi: 10.3389/fphar.2024.1341039 (PMC11070777; doi:10.3389/fphar.2024.1341039)
Supplement: Supplementary file 11 [file DataSheet2.ZIP › GSEA/GO_CC/my_analysis.Gsea.1599461408869/GO_LATE_ENDOSOME.html]

Details for gene set GO\_LATE\_ENDOSOME[GSEA]

|  || Dataset | filtered\_dataset.sample\_info.cls#WT\_versus\_NCAPD3\_MUT |
| Phenotype | sample\_info.cls#WT\_versus\_NCAPD3\_MUT |
| Upregulated in class | WT |
| GeneSet | GO\_LATE\_ENDOSOME |
| Enrichment Score (ES) | 0.32999763 |
| Normalized Enrichment Score (NES) | 1.3603158 |
| Nominal p-value | 0.118421055 |
| FDR q-value | 0.6006424 |
| FWER p-Value | 1.0 |
Table: GSEA Results Summary

  

Fig 1: Enrichment plot: GO\_LATE\_ENDOSOME      
 Profile of the Running ES Score & Positions of GeneSet Members on the Rank Ordered List

  

| SYMBOL | TITLE | RANK IN GENE LIST | RANK METRIC SCORE | RUNNING ES | CORE ENRICHMENT || 1 | 1497 | CTNS | 58 | 0.883 | 0.0392 | Yes |
| 2 | 22879 | MON1B | 151 | 0.735 | 0.0403 | Yes |
| 3 | 55240 | STEAP3 | 169 | 0.710 | 0.0931 | Yes |
| 4 | 55745 | AP5M1 | 246 | 0.634 | 0.0965 | Yes |
| 5 | 353376 | TICAM2 | 271 | 0.617 | 0.1357 | Yes |
| 6 | 538 | ATP7A | 305 | 0.588 | 0.1658 | Yes |
| 7 | 79443 | FYCO1 | 313 | 0.586 | 0.2144 | Yes |
| 8 | 84946 | LTV1 | 319 | 0.583 | 0.2642 | Yes |
| 9 | 55697 | VAC14 | 329 | 0.576 | 0.3104 | Yes |
| 10 | 1183 | CLCN4 | 373 | 0.551 | 0.3300 | Yes |
| 11 | 55317 | AP5S1 | 573 | 0.437 | 0.2270 | No |
| 12 | 23274 | CLEC16A | 629 | 0.408 | 0.2248 | No |
| 13 | 404636 | FAM45A | 764 | 0.341 | 0.1597 | No |
| 14 | 3949 | LDLR | 929 | -0.353 | 0.0742 | No |
| 15 | 4864 | NPC1 | 964 | -0.384 | 0.0850 | No |
| 16 | 27074 | LAMP3 | 1158 | -0.522 | -0.0059 | No |
| 17 | 23461 | ABCA5 | 1206 | -0.583 | 0.0136 | No |
| 18 | 1956 | EGFR | 1266 | -0.657 | 0.0314 | No |
| 19 | 1649 | DDIT3 | 1341 | -0.779 | 0.0496 | No |
Table: GSEA details [plain text format]

  

Fig 2: GO\_LATE\_ENDOSOME      
 Blue-Pink O' Gram in the Space of the Analyzed GeneSet

  

Fig 3: GO\_LATE\_ENDOSOME: Random ES distribution      
 Gene set null distribution of ES for **GO\_LATE\_ENDOSOME**
